# Case Report: A 91-Year-Old Patient With Non-Small Cell Lung Cancer Harboring MET Y1003S Point Mutation

**DOI:** 10.3389/fmed.2021.772998

**Published:** 2022-01-31

**Authors:** Beili Gao, Ran Zeng

**Affiliations:** ^1^Department of Pulmonary and Critical Care Medicine, Ruijin Hospital, Shanghai Jiao Tong University School of Medicine, Shanghai, China; ^2^Institute of Respiratory Diseases, Shanghai Jiao Tong University School of Medicine, Shanghai, China; ^3^Shanghai Key Laboratory of Emergency Prevention, Diagnosis and Treatment of Respiratory Infectious Diseases, Shanghai, China

**Keywords:** MET Y1003 mutation, crizotinib, non-small cell lung cancer, next-generation sequencing, case report

## Abstract

**Background:**

The Y1003S point mutation in exon 14 of mesenchymal-epithelial transition (MET) is a rare mutation that can lead to oncogenic transformation. Few data are available on the characteristics of this mutation. This report presents an elderly patient with non-small cell lung cancer (NSCLC) and a Y1003S mutation in MET detected by next-generation sequencing (NGS).

**Case Report:**

In October 2020, a 91-year-old male was admitted to the Department of Respiratory and Critical Care Medicine, Ruijin Hospital because of an increased carcinoembryonic antigen. Imaging revealed highly suspicious lesions in the right upper lobe of the lung, right apex, and left upper lobe with traction of the adjacent pleura. The patient was histologically confirmed as having adenocarcinoma and the MET Y1003S mutation was detected by the NGS subsequently. After evaluation, the patient started crizotinib treatment in December 2020. In the first assessment of tumor response, a chest CT scan in January 2021 showed a partial response. The patient experienced a pulmonary embolism and an abnormal liver function during the treatment and recovered after symptomatic treatment. He maintained a partial response in the last available assessment in July 2021, with the right upper lung lesion being 26 × 9 mm.

**Conclusion:**

The MET Y1003S mutation was detected in this case, and the patient achieved a partial response using crizotinib. This case highlighted the role of NGS in detecting a rare mutation. Successful remission of complications in such an elderly patient necessitates careful and timely management.

## Introduction

Lung cancer is the leading cause of cancer mortality globally (1). The hepatocyte growth factor (HGF)/mesenchymal-epithelial transition factor (MET) axis plays an important role in the lung cancer development, progression, and resistance to therapies (2–4). Patients with non-small cell lung cancer (NSCLC), harboring high MET amplification or mutations in exon 14 of MET, show high responses to MET inhibitors (5), and guidelines suggest the use of crizotinib in such patients (6–8). Nevertheless, different MET mutations may display different behaviors (i.e., response or resistance) to MET inhibitors (8, 9). The Y1003 residue is encoded by exon14 of the MET gene. It is located in the binding site for the E3 ubiquitin ligase c-Cbl, which is involved in the ubiquitination and degradation of MET. The Y1003 point mutations can lead to oncogenic transformation *in vitro* and were reported in lung cancer (10).

A phase I trial of crizotinib in patients with an advanced NSCLC harboring exon 14 mutations in MET showed an acceptable profile and a preliminary efficacy (11). However, little data are available about the response of tumors harboring the Y1003S point mutation to MET-targeted therapies. Therefore, this report presents an elderly patient with NSCLC and Y1003S mutation in MET treated with crizotinib.

## Case Presentation

**Figure 1:**
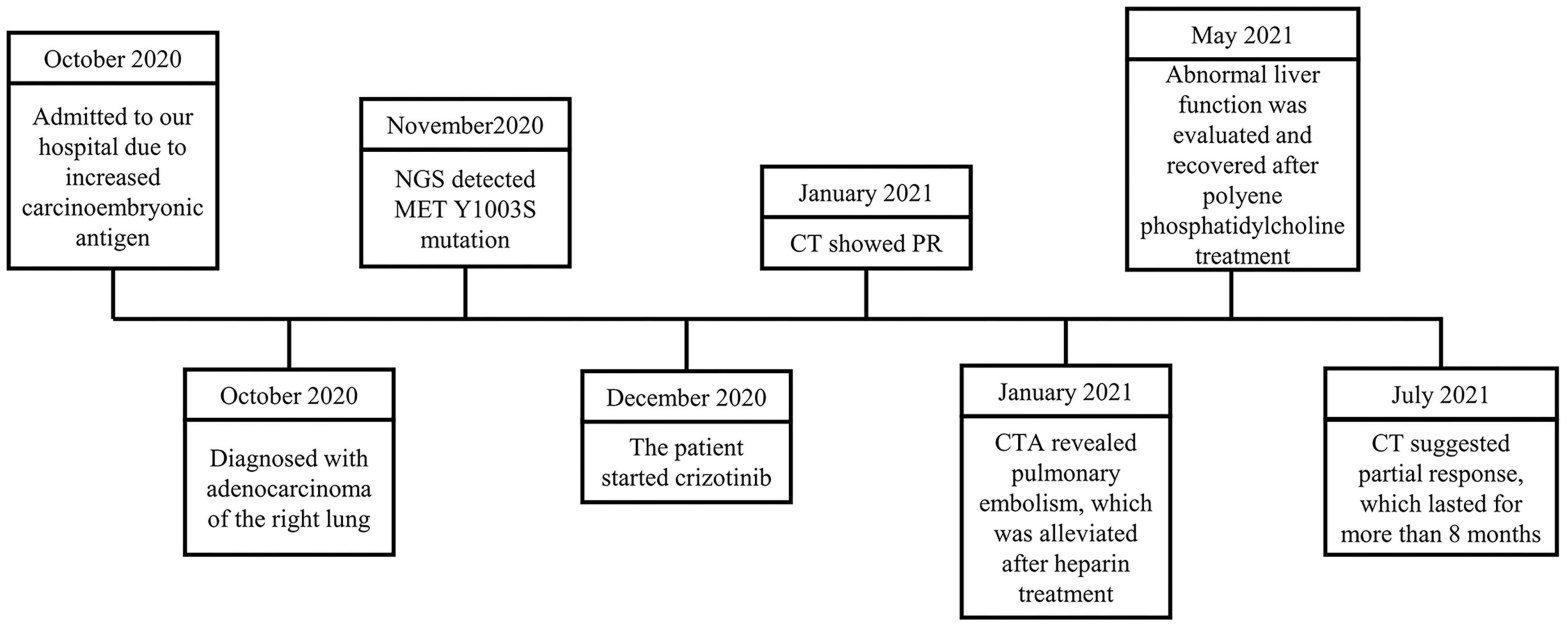
Timeline of the patient.

A 91-year-old male was admitted to the Department of Respiratory and Critical Care Medicine, Ruijin Hospital in October 2020 because of an incidental finding of a lung mass for 20 days, increased carcinoembryonic antigen (CEA) levels, and localized emphysema in a local hospital (Figure 1). The patient first consulted at the local hospital because of cough, shortness of breath, anorexia, fever, chest pain, and other respiratory symptoms with unknown causes for more than 3 months. For further diagnosis, the patient was admitted to our hospital. He denied a smoking and drinking history, but both parents died of lung cancer. The patient had coronary heart disease (taking aspirin), hypertension (taking candesartan cilexetil), lacunar cerebral infarction, colon polyps (tubular adenoma with low-grade intraepithelial neoplasia revealed by colonoscopic polypectomy), and a history of tuberculosis 60 years ago.

**Figure 2:**
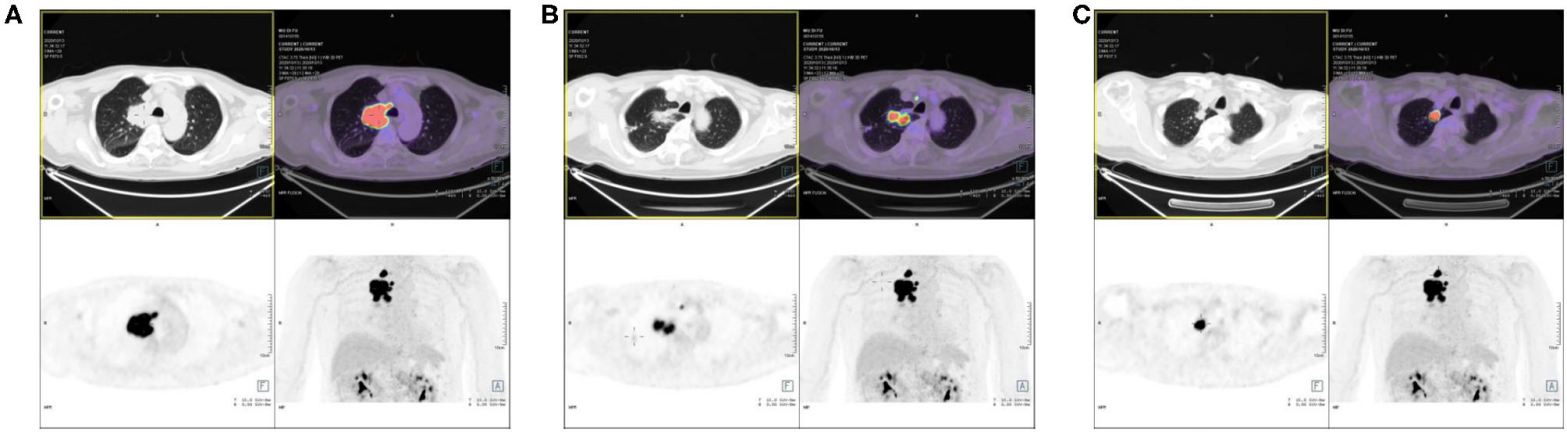
Positron emission tomography (PET)/MRI of the patient. **(A)** The high metabolic mass in the upper right lobe. **(B)** The high metabolic nodule at the apex of the right lung. **(C)** The small nodules in the upper right lobe with traction of the adjacent pleura, with slightly increased metabolism.

Positron emission tomography (PET)—MRI of the chest revealed a highly metabolic mass in the upper right lobe, a highly metabolic nodule at the apex of the right lung, and small nodules in the upper right lobe with traction of the adjacent pleura, with a slightly increased metabolism (Figure 2). Furthermore, adenocarcinoma cells were found by right upper lobe lesion biopsy. After subsequent evaluation, he was diagnosed with stage T3N2M0 adenocarcinoma of the right lung.

**Figure 3:**
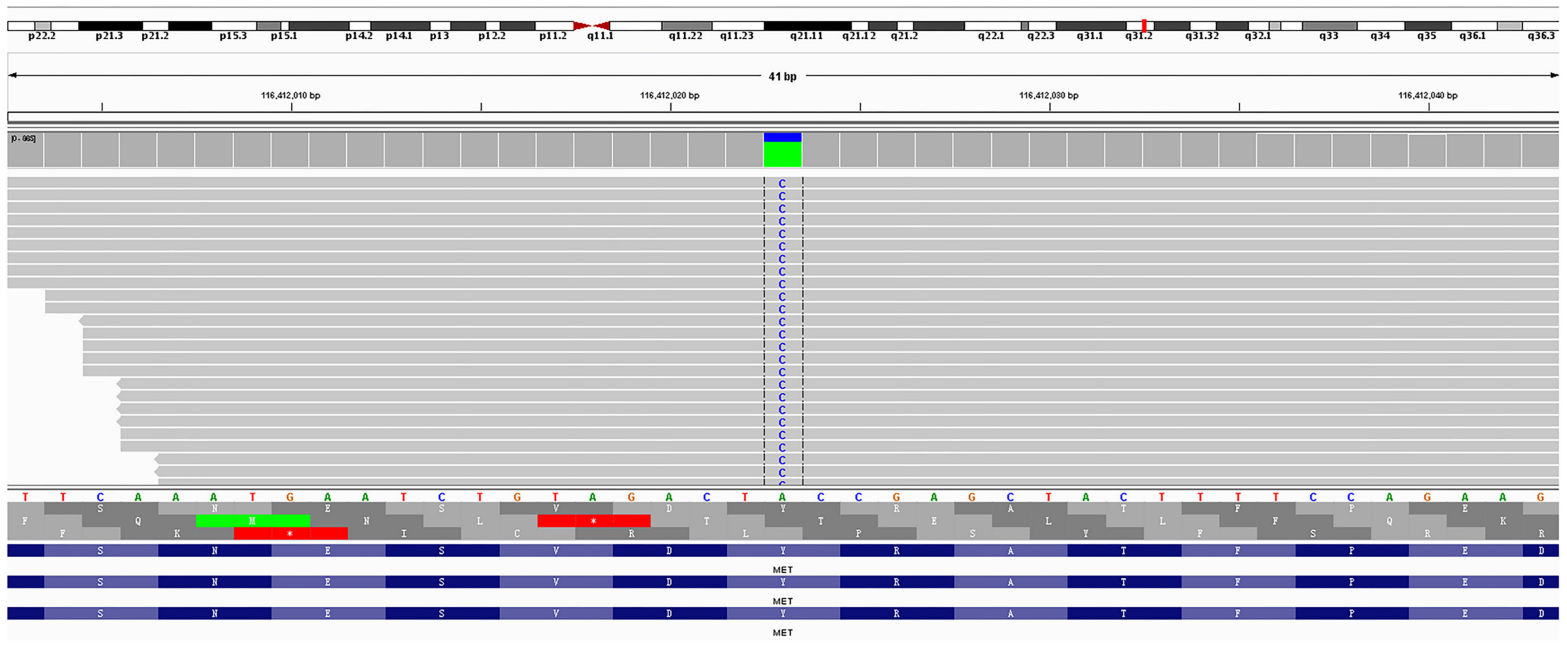
Genetic analysis showing the Y1003S point mutation.

**Figure 4:**
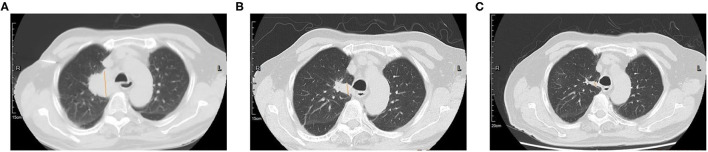
Computed tomography (CT) scan of the primary lung lesion before and after crizotinib treatment. **(A)** In November 2020, the tumor size was 55 × 43 mm; **(B)** In January 2021, the tumor size was 26 × 17 mm; **(C)** In May 2021, the tumor size was 27 × 9 mm.

Amplification-refractory mutation system (ARMS)-polymerase chain reaction (PCR)-based single gene tests for EGFR, ALK, ROS1, BRAF, NRAS, KRAS, ERBB2, PIK3CA, and MET exon 14 skipping were all negative in the tissue biopsy. Then, rare mutations were tested by next-generation sequencing (NGS) for tissue biopsy (F1CDX; Foundation Medicine, Massachusetts, United States) and liquid biopsy (America Geneseeq Prime, Nanjing, China). An MET Y1003S mutation was detected in the tissue (Figure 3) but not in the liquid biopsy. There were no EGFR mutations, MET amplifications, or ALK/ROS1 fusion events in both testing. The patient started crizotinib 250 mg b.i.d. on December 20, 2020. Chest CT scan on January 11, 2021 showed a partial response and significant improvement of the lung lesions (right upper lung: from 55 × 43 mm to 23 × 17 mm, Figure 4).

After 1 month of crizotinib treatment, the patient was admitted because of chest distress, which could be alleviated by rest. Pulmonary artery thin layer CTA revealed pulmonary embolism in the left lower lobe. He was treated with low-molecular-weight heparin subcutaneous injection 0.4 ml q 12 h for 3 days and q.d. for 4 days, and then rivaroxaban 10 mg/day. His symptoms were alleviated after the treatment. Drug-induced abnormal liver function was considered because of the increased ALT and AST. The patient was given polyene phosphatidylcholine 228 mg two tablets t.i.d. On May 21, liver function was recovered. On July 30, 2021, the right upper lung lesion was 26 mm × 9 mm, which suggested a partial response.

## Discussion

Since Y1003 mutations are predicted to be similar to MET exon 14 skipping in functionality (12, 13), crizotinib was proposed to patients with advanced NSCLC harboring Y1003S MET mutation. The patient displayed a favorable response to crizotinib that lasted for more than 8 months.

Tests for EGFR, ALK, RET, etc., are recommended by the NSCLC guidelines of Chinese Society of Clinical Oncology (14). Indeed, in China, ARMS-PCR test kits are reimbursed, but not the next-generation sequencing. Furthermore, the high cost and lack of targeted therapy for some mutations restrict the application of NGS. In the case reported here, next-generation sequencing was performed after PCR found no mutations, and MET Y1003S mutation was found in the tissue biopsy. The CSCO guidelines also emphasize the importance of tissue biopsy. In the case of limited biopsy tissue or only enough for repeated test, large-panel NGS should be considered as the first choice. It can avoid missing rare mutations in single-gene or small-panel testing, and it is more conducive to timing the diagnosis with the treatment. As shown in this case, NGS can detect rare mutations and, thus, provide evidence for patient treatment. Besides, the type of sample also appears to be an issue. In this case, the mutation was found in the tumor itself and not in the liquid biopsy. Significantly, liquid biopsy shows importance when it is difficult to obtain tissue samples, especially when a second biopsy is not feasible after drug resistance.

Furthermore, this patient was elderly and had several comorbidities. Chemotherapy and immunotherapy may not be suitable for such patients because of poor tolerance. Treatments seriously affect their quality of life and have potential risks. Clarification of drug targets by next-generation sequencing is of great significance for improving the quality of life of elderly patients and improving the prognosis. In the case reported here, pulmonary embolism occurred during crizotinib treatment. Common adverse reactions to crizotinib include visual abnormalities (55%), nausea (51%), vomiting (46%), diarrhea (46%), edema (39%), constipation (38%), and fatigue (26%) (15). In addition, study A8081007 reported pulmonary embolism (3.5%) and dyspnea (2.3%) with crizotinib (16). Still, it is unknown whether pulmonary embolism was an adverse drug reaction to crizotinib or a complication of the lung cancer itself.

The receptor tyrosine kinase MET is an important regulator of cell growth, regeneration, and development (2–4). Many alterations in MET have been reported in various human cancers such as small cell lung cancer and NSCLC (5). The Y1003C amino acid substitution was detected in a patient with NSCLC, and this mutation might be the main reason for MET overexpression (17). In addition, Y1003F mutation had been shown to convert the receptor into a transforming protein in the absence of a ligand (18). These data show that Y1003 mutations might lead to oncogenic transformation and would be an actionable site for targeted treatment. The case reported here strongly supports that Y1003S point mutation in lung cancer could have a response to crizotinib, as suggested by a previous case report (12). Patients with MET amplification show favorable response to crizotinib (19). The NSCLC with MET and other mutations (e.g., D1010H and METex14) also appears to respond to crizotinib (20–22). Drilon et al. (22) reported an objective response rate (ORR) of 32% in patients with NSCLC and MET exon 14 alterations. In patients with MET amplification, the ORR was 14.3-38.1% (19). Further studies are needed to validate the sensitivity of various Y1003 to MET inhibitors.

For MET exon-skipping mutations, in addition to crizotinib, camatinib and sevotinib are also available. Crizotinib was approved in 2013 for ALK-positive NSCLC in China, and articles are reporting the use of crizotinib in patients with MET mutation. Camatinib (23) and sevotinib (ClinicalTrials.org NCT02897479) were all approved in 2021 in China and were not available when the reported case was treated. Therefore, considering the high requirements for drug safety due to advanced age and comorbidities and drug accessibility in China, crizotinib was given to this patient. Still, future studies could examine camatinib and sevotinib in such cases of MET mutations.

## Conclusion

In conclusion, response to crizotinib was observed in this case of NSCLC with Y1003S MET mutation, which lasted for more than 8 months. This case demonstrates that the MET inhibitor crizotinib shows an antitumor response to Y1003S MET mutation, which might be a new targetable mutation in NSCLC. Besides, this case indicated that NGS shows a beneficial effect on detecting a rare mutation. Successful remission of complications in such an elderly patient requires a careful and timely management.

## Data Availability Statement

The original contributions presented in the study are included in the article/Supplementary Material, further inquiries can be directed to the corresponding author/s.

## Ethics Statement

The authors certify that they have obtained all appropriate patient consent forms. In the form, the patient has given his consent for his images and other clinical information to be reported in the journal. The patient understands that his name and initials will not be published and due efforts will be made to conceal his identity, but anonymity cannot be guaranteed. The patients/participants provided their written informed consent to participate in this study.

## Author Contributions

BG contributed to the conception, design, interpretation of data, and drafted and critically revised the manuscript. RZ contributed to data acquisition, analysis of data, and drafting of the manuscript. All authors read and approved the final version of the manuscript.

## Conflict of Interest

The authors declare that the research was conducted in the absence of any commercial or financial relationships that could be construed as a potential conflict of interest.

## Publisher's Note

All claims expressed in this article are solely those of the authors and do not necessarily represent those of their affiliated organizations, or those of the publisher, the editors and the reviewers. Any product that may be evaluated in this article, or claim that may be made by its manufacturer, is not guaranteed or endorsed by the publisher.
